# Supplementary material for: HIVprotI: an integrated web based platform for prediction and design of HIV proteins inhibitors
Source: J Cheminform. 2018 Mar 9;10:12. doi: 10.1186/s13321-018-0266-y (PMC5845081; doi:10.1186/s13321-018-0266-y)
Supplement: Supplementary file 2 — Additional file 2. Source code of HIVProtI web server. [file 13321_2018_266_MOESM2_ESM.zip › HIVprotI_Source-code/hivprotI/weka-3-6-6/doc/weka/classifiers/functions/PLSClassifier.html]

PLSClassifier


---


|  |  |  |
| --- | --- | --- |
| **Overview** \| **Package** \| **Class** \| **Tree** \| **Deprecated** \| **Index** \| **Help** \| **Weka's home** | |  |
| **PREV CLASS**   **NEXT CLASS** | **FRAMES**    **NO FRAMES**     **All Classes** |  |
| SUMMARY: NESTED \| FIELD \| CONSTR \| METHOD | DETAIL: FIELD \| CONSTR \| METHOD |  |


---


## weka.classifiers.functions Class PLSClassifier

```
java.lang.Object
  weka.classifiers.Classifier
      weka.classifiers.functions.PLSClassifier
```

**All Implemented Interfaces:**: java.io.Serializable, java.lang.Cloneable, CapabilitiesHandler, OptionHandler, RevisionHandler

---

``` public class PLSClassifier extends Classifier ```

A wrapper classifier for the PLSFilter, utilizing the PLSFilter's ability to perform predictions.


Valid options are:

```
 -filter <filter specification>
  The PLS filter to use. Full classname of filter to include,  followed by scheme options.
  (default: weka.filters.supervised.attribute.PLSFilter)
```

```
 -D
  If set, classifier is run in debug mode and
  may output additional info to the console
```

```
 Options specific to filter weka.filters.supervised.attribute.PLSFilter ('-filter'):
```

```
 -D
  Turns on output of debugging information.
```

```
 -C <num>
  The number of components to compute.
  (default: 20)
```

```
 -U
  Updates the class attribute as well.
  (default: off)
```

```
 -M
  Turns replacing of missing values on.
  (default: off)
```

```
 -A <SIMPLS|PLS1>
  The algorithm to use.
  (default: PLS1)
```

```
 -P <none|center|standardize>
  The type of preprocessing that is applied to the data.
  (default: center)
```

**Version:**
:   $Revision: 1.4 $

**Author:**
:   fracpete (fracpete at waikato dot ac dot nz)

**See Also:**: Serialized Form

---

**Constructor Summary**

`PLSClassifier()`


| **Method Summary** | |
| --- | --- |
| `void` | `buildClassifier(Instances data)`             builds the classifier |
| `double` | `classifyInstance(Instance instance)`             Classifies the given test instance. |
| `java.lang.String` | `filterTipText()`             Returns the tip text for this property |
| `Capabilities` | `getCapabilities()`             Returns default capabilities of the classifier. |
| `Filter` | `getFilter()`             Get the PLS filter. |
| `java.lang.String[]` | `getOptions()`             returns the options of the current setup |
| `java.lang.String` | `getRevision()`             Returns the revision string. |
| `java.lang.String` | `globalInfo()`             Returns a string describing classifier |
| `java.util.Enumeration` | `listOptions()`             Gets an enumeration describing the available options. |
| `static void` | `main(java.lang.String[] args)`             Main method for running this classifier from commandline. |
| `void` | `setFilter(Filter value)`             Set the PLS filter (only used for setup). |
| `void` | `setOptions(java.lang.String[] options)`             Parses the options for this object. |
| `java.lang.String` | `toString()`             returns a string representation of the classifier |

**Methods inherited from class weka.classifiers.Classifier**

`debugTipText, distributionForInstance, forName, getDebug, makeCopies, makeCopy, setDebug`

**Methods inherited from class java.lang.Object**

`equals, getClass, hashCode, notify, notifyAll, wait, wait, wait`

**Constructor Detail**

### PLSClassifier

```
public PLSClassifier()
```


**Method Detail**

### globalInfo

```
public java.lang.String globalInfo()
```

:   Returns a string describing classifier

    :   **Returns:**: a description suitable for displaying in the explorer/experimenter gui

---


### listOptions

```
public java.util.Enumeration listOptions()
```

:   Gets an enumeration describing the available options.

    :   **Specified by:**: `listOptions` in interface `OptionHandler` **Overrides:**: `listOptions` in class `Classifier`
    :   **Returns:**: an enumeration of all the available options.

---


### getOptions

```
public java.lang.String[] getOptions()
```

:   returns the options of the current setup

    :   **Specified by:**: `getOptions` in interface `OptionHandler` **Overrides:**: `getOptions` in class `Classifier`
    :   **Returns:**: the current options

---


### setOptions

```
public void setOptions(java.lang.String[] options)
                throws java.lang.Exception
```

:   Parses the options for this object.


    Valid options are:

    ```
     -filter <filter specification>
      The PLS filter to use. Full classname of filter to include,  followed by scheme options.
      (default: weka.filters.supervised.attribute.PLSFilter)
    ```

    ```
     -D
      If set, classifier is run in debug mode and
      may output additional info to the console
    ```

    ```
     Options specific to filter weka.filters.supervised.attribute.PLSFilter ('-filter'):
    ```

    ```
     -D
      Turns on output of debugging information.
    ```

    ```
     -C <num>
      The number of components to compute.
      (default: 20)
    ```

    ```
     -U
      Updates the class attribute as well.
      (default: off)
    ```

    ```
     -M
      Turns replacing of missing values on.
      (default: off)
    ```

    ```
     -A <SIMPLS|PLS1>
      The algorithm to use.
      (default: PLS1)
    ```

    ```
     -P <none|center|standardize>
      The type of preprocessing that is applied to the data.
      (default: center)
    ```

    :   **Specified by:**: `setOptions` in interface `OptionHandler` **Overrides:**: `setOptions` in class `Classifier`
    :   **Parameters:**: `options` - the options to use **Throws:**: `java.lang.Exception` - if setting of options fails

---


### filterTipText

```
public java.lang.String filterTipText()
```

:   Returns the tip text for this property

    :   **Returns:**: tip text for this property suitable for displaying in the explorer/experimenter gui

---


### setFilter

```
public void setFilter(Filter value)
               throws java.lang.Exception
```

:   Set the PLS filter (only used for setup).

    :   **Parameters:**: `value` - the kernel filter. **Throws:**: `java.lang.Exception` - if not PLSFilter

---


### getFilter

```
public Filter getFilter()
```

:   Get the PLS filter.

    :   **Returns:**: the PLS filter

---


### getCapabilities

```
public Capabilities getCapabilities()
```

:   Returns default capabilities of the classifier.

    :   **Specified by:**: `getCapabilities` in interface `CapabilitiesHandler` **Overrides:**: `getCapabilities` in class `Classifier`
    :   **Returns:**: the capabilities of this classifier **See Also:**: `Capabilities`

---


### buildClassifier

```
public void buildClassifier(Instances data)
                     throws java.lang.Exception
```

:   builds the classifier

    :   **Specified by:**: `buildClassifier` in class `Classifier`
    :   **Parameters:**: `data` - the training instances **Throws:**: `java.lang.Exception` - if something goes wrong

---


### classifyInstance

```
public double classifyInstance(Instance instance)
                        throws java.lang.Exception
```

:   Classifies the given test instance. The instance has to belong to a
    dataset when it's being classified.

    :   **Overrides:**: `classifyInstance` in class `Classifier`
    :   **Parameters:**: `instance` - the instance to be classified **Returns:**: the predicted most likely class for the instance or Instance.missingValue() if no prediction is made **Throws:**: `java.lang.Exception` - if an error occurred during the prediction

---


### toString

```
public java.lang.String toString()
```

:   returns a string representation of the classifier

    :   **Overrides:**: `toString` in class `java.lang.Object`
    :   **Returns:**: a string representation of the classifier

---


### getRevision

```
public java.lang.String getRevision()
```

:   Returns the revision string.

    :   **Specified by:**: `getRevision` in interface `RevisionHandler` **Overrides:**: `getRevision` in class `Classifier`
    :   **Returns:**: the revision

---


### main

```
public static void main(java.lang.String[] args)
```

:   Main method for running this classifier from commandline.

    :   **Parameters:**: `args` - the options


---


|  |  |  |
| --- | --- | --- |
| **Overview** \| **Package** \| **Class** \| **Tree** \| **Deprecated** \| **Index** \| **Help** \| **Weka's home** | |  |
| **PREV CLASS**   **NEXT CLASS** | **FRAMES**    **NO FRAMES**     **All Classes** |  |
| SUMMARY: NESTED \| FIELD \| CONSTR \| METHOD | DETAIL: FIELD \| CONSTR \| METHOD |  |


---
